# Multifocal early gastric cancer in a patient with atrophic gastritis and pernicious anemia

**DOI:** 10.1016/j.ijscr.2020.06.010

**Published:** 2020-06-12

**Authors:** Tommaso Zurleni, Michele Altomare, Giovanni Serio, Filippo Catalano

**Affiliations:** a Department of General Surgery, ASST Valle Olona, Busto Arsizio Hospital, Italy; b Department of Anatomical Pathology, ASST Valle Olona, Busto Arsizio Hospital, Italy; c Emergency Endoscopy Unit, Borgo Trento Hospital, Verona, Italy

**Keywords:** Multifocal early gastric cancer, Pernicious anemia, Autoimmune metaplastic atrophic gastritis, Subtotal gastrectomy

## Abstract

**Introduction:**

Pernicious anemia (PA) caused by vitamin B12 deficiency is associated with Autoimmune Metaplastic Atrophic Gastritis (AMAG). Patients with AMAG have threefold risk of the development of gastric cancer.

**Presentation of case:**

We describe a case of a 66 year old man with a history of PA and atrophic antral-corpus gastritis. After endoscopic and chromoendoscopic evaluation the patient was treated with subtotal gastrectomy plus D2 lymphadenectomy. The tumor was diagnosed as Stage Ia; pT1a pN0 pM0 G2 with multiple foci of high grade dysplasia and intramucosal adenocarcinoma.

**Discussion:**

Multifocal Early Gastric Cancer can be a problem for minimally invasive treatment such as endoscopic excision.

Surgical management where it is not possible Endoscopic Mucosal Resection or Submucosal Resection (EMR/ESD) should include D1 or more type of lymphadenectomy because of the risk of nodes metastases.

The chromoendoscopic evaluation may be helpful in the preoperative work-up and during the follow-up period.

**Conclusion:**

Multidisciplinary approach is very important to reduce the under-treatment risk in multifocal early gastric cancer. Further studies will be needed to evaluate the safety of Subtotal vs Total Gastrectomy in this kind of disease.

## Highlights

• Autoimmune Metaplastic Atrophic Gastritis and Pernicious Anemia increase the risk of cancer.
• Multifocality is a condition described in 0.8–22% of Early Gastric Cancer.
• Subtotal gastrectomy can be safely performed when endoscopic treatment is not feasible.
• Multidisciplinary approach is very important to reduce the under-treatment risk.

## 1 Introduction

### 1.1 Autoimmune metaplastic atrophic gastritis (AMAG) and pernicious anemia (PA)

This case-report has been reported in line with the SCARE criteria [1].

AMAG is the result of antibody-mediated destruction of parietal cells that leads to long-term hematologic and neurologic consequences like iron deficiency anemia, Pernicious Anemia (PA), depression, irritability and pshychosis [2].

The target antigens are the parietal cell H^+^-K^+^ ATPase, and Intrinsic Factor [3].

Historically, atrophic gastritis has been broadly divided into environmental and autoimmune etiologies [4]; the differences between Type A (AMAG) and Type B (HP Infection) gastritis, are summarized in Table 1.

**Table 1:** Type A (AMAG) and Type B (HP Infection) gastritis differences.

	Autoimmune	Infectious
Type	A	B
Mechanism	Autoantibodies against parietal cell antigens	HP infection
Distribution	Body	Antrum
Endoscopic Findings	Early disease: minimal findings Late disease: body predominant atrophy (pseudopolyps)	Antral predominant or multifocal inflammation or atrophy
Risk of malignancy	Epithelial dysplasia, adenomas, adenocarcinoma. Type I endocrine tumor	Epithelial dysplasia and carcinoma

Approximately 20–30% of patient with iron deficiency anemia without clinical evidence of blood loss have been reported to have AMAG [5]. PA is rarer and is a result of advanced AMAG. In a Swedish cohort study of more than 4000 patients with PA followed for 20 years, there was a 3-fold increased risk of gastric carcinoma and 13-fold increased risk of gastric carcinoid [6]. A more recent cohort study has reported an annual incidence rate of 1.36% person-year for gastric neoplastic lesion and 0.25% for gastric cancer (GC) [7]. Therefore, in the last years, reliable data are emerging that PA is linked to increased risk of Gastric Cancer [8]. According to these data some studies suggest that PA should be considered as independent risk factor able to target AMAG-patients subgroup with higher risk of neoplasm in Italy [9].

### 1.2 Multifocal early gastric cancer

Despite the reported declining incidence, GC is one of the most common causes of cancer mortality worldwide [10]. Different epidemiological trends in the intestinal type (InT) and diffuse type (DiT) Lauren histotypes have also been observed. The declining incidence of GC has been linked to the decreasing number of InT; on the other hand, the incidence of DiT is generally stable [11].

The term “Early Gastric Cancer” defined in 1971 by the Japanese Society of Gastroenterology and Endoscopy as carcinoma limited to gastric mucosa and/or submucosa, regardless of lymph node status, has continued to leave controversies over the years [12]. The percentage of lymph node metastases get from literature in EGC is still high: 11% for InT and 25.4% for DiT [13,14].

## 2 Presentation of case

We report a case of a 66-year old man with a history of 6 years of pernicious anemia and detection of atrophic antral-corpus gastritis.

Antibodies against intrinsic factor and anti-parietal cell were positive.

**Fig. 1:**
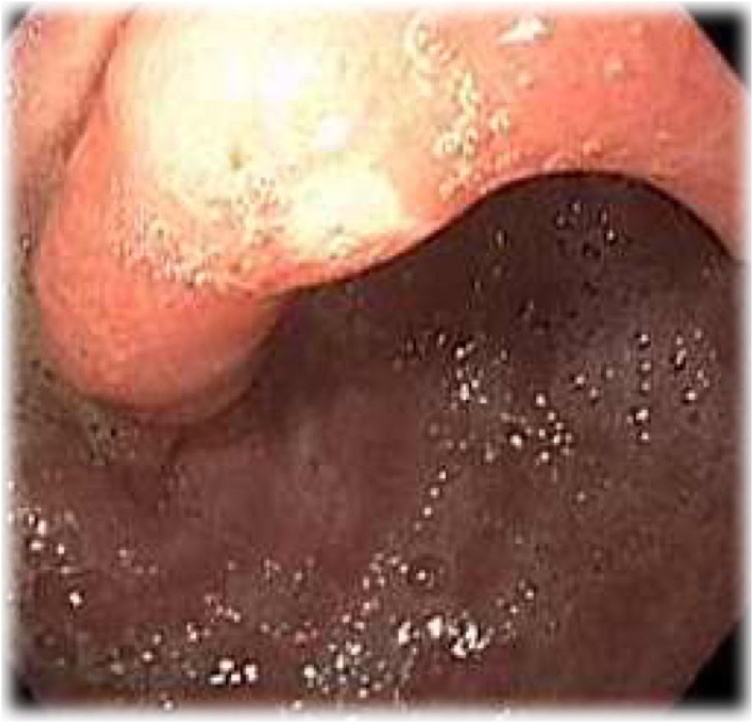
Primary endoscopical evaluation.

During annual endoscopy control, it was found a small size distal lesion of the antral area of the stomach (Fig. 1).

Histological examination revealed a high-grade dysplasia (Hp neg with expression of Ki67 and P53 in more than 95% of cells).

**Fig. 2:**
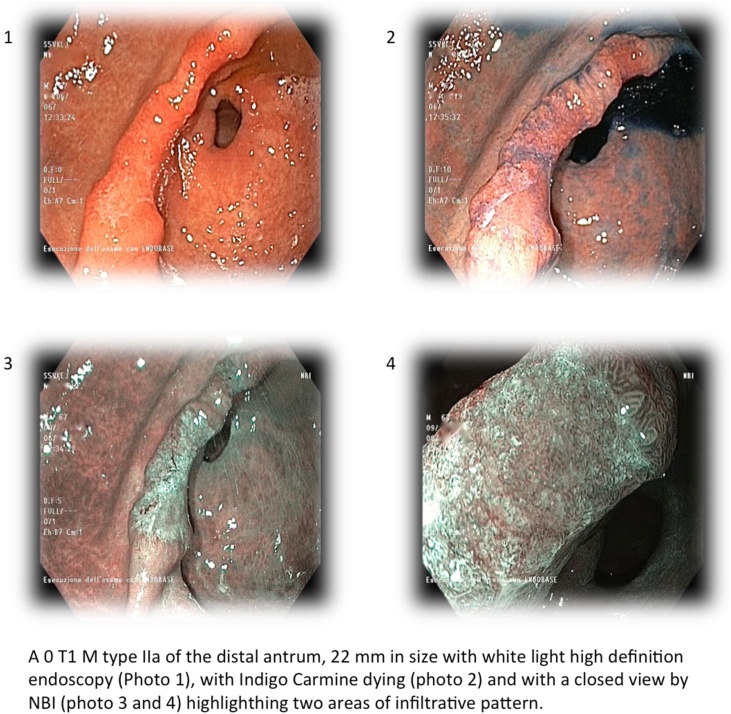
Chromoendoscopy control.

Therefore the patient underwent chromoendoscopy and new biopsies of the superficial elevated pre-pyloric lesion of 22 mm (macroscopic evaluation: Type 0 T1m IIa) (Fig. 2).

Histological examination showed an intestinal type adenocarcinoma by Lauren. CT scan excluded distant metastases and lymphadenopathy, CEA: 3.8 and CA19.9: 4.

We decided to perform surgery after a multidisciplinary group discussion of the clinical case.

**Fig. 3:**
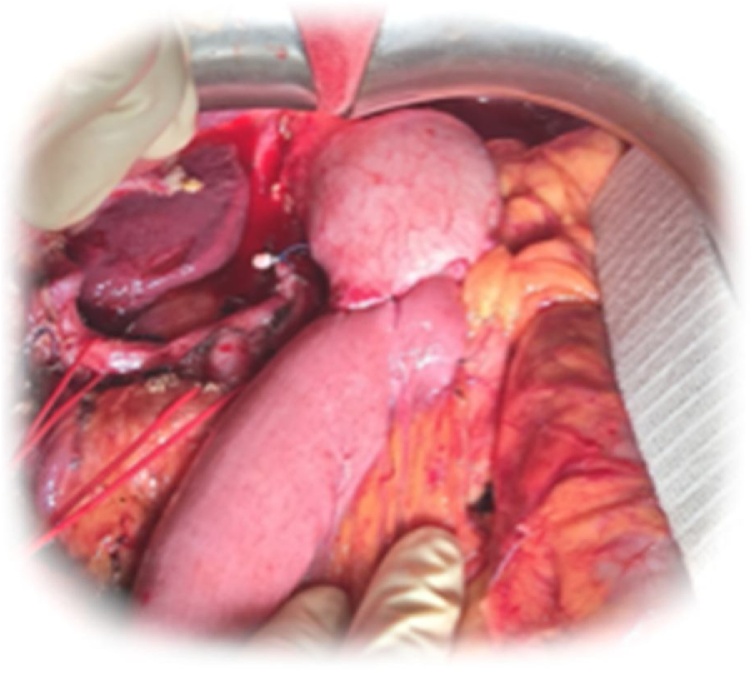
D2 Subtotal gastrectomy.

The patient underwent open subtotal gastrectomy with D2 type of lymphadenectomy (stat. n° 1-3-4-5-6-7-8(a,p)-9-11p-12(a,b,p)). Gastro-jejunal circular mechanical anastomosis with a Roux-en-Y type of reconstruction was performed (Fig. 3).

Neither intra nor post-operative complication occurred.

**Fig. 4:**
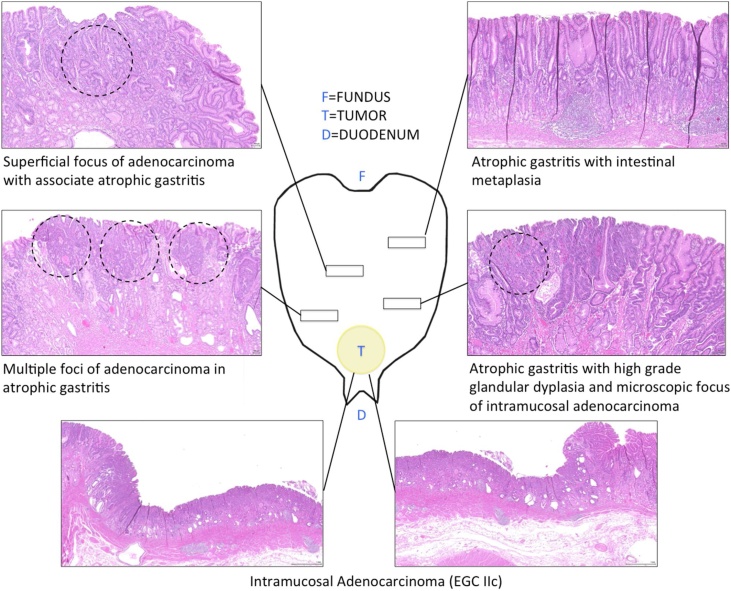
Pathological mapping of the specimen.

Histological examination revealed Multifocal Early Gastric Cancer infiltrating the muscolaris mucosae; intestinal type by Lauren, tubular type by WHO moderately differentiated (Fig. 4).

Cytology on peritoneal washing was negative. 48 negative nodes were retrieved (pT1aN0M0 G2).

He had an uneventful recovery and he was discharged from the hospital 11 POD.

### 2.1 Follow-up

The patient underwent regular follow-up every 6 months by standard clinical and radiological controls and by endoscopic/chromoendoscopic evaluation. 5 years after surgery the patient is free from disease.

## 3 Discussion

Multifocality is a condition described in 0.8–22% of EGC [15] and can be a problem for minimally invasive treatment such as endoscopic excision moreover in western countries where endoscopic techniques are not standardized such as in the Eastern hospital. In a recent study Suzuki et al. [16] evaluate the outcomes of EGC patients after non curative ESD and define the necessity of following studies to identify patients who can underwent additional surgery, considering the risk of recurrence and metastasis.

An adequate preoperative investigation by endoscopy and chromoendoscopy and multidisciplinary approach are very important to plan the best therapeutic strategy in order to reduce the under-treatment risk, mostly considering that the sensitivity (62%) and specificity (65.7%) of CT-scan also in high grade tumors are still not trustworthy as showed in a recent study from Fukagawa et al. [17].

Interestingly, a recent study on genetic pathways of multiple intramucosal gastric cancer, demonstrated that synchronously developed multiple early gastric cancer shared the common feature of the MSI (microsatellite instability)/MSS (microsatellite stable) phenotype [18].

Regarding the prognosis, even if Kim et al. [19] concluded that synchronous multifocality of EGC does not increase the risk of lymph node metastases compared with solitary EGC, there are a lot of several studies in literature that identify multifocality as independent risk factor for developing Metachronous Gastric Cancer [20]. Moreover in a recent Cohort study Gertler analysed the prevalence of lymph node metastases in a group of 793 patients with early esophageal and gastric cancer, showing a lymph node involvement in 12.6%, with a different overall survival (OS) between the two groups (N0 89%; N+ 69%) [21].

Japanese Gastric Cancer Treatment guidelines (ver. 4) suggest in case of cT1N+ tumors a D2 lymphadenectomy, and D1 and D1+ lymphadenectomy in T1a tumors that do not meet criteria for EMR/ESD, and in all cT1bN0 [22]. Moreover Morgagni et al. [23] show how subtotal gastrectomy is strongly recommended despite of gastrectomy, when EMR/ESD approach is not faithful, for several reason: 1) secondary lesion are generally site in lower third near the main lesion; 2) secondary ECG sited in the upper third are rare; 3) subtotal gastrectomy have lower morbidity and mortality, a better quality of life and a similar survival rate compared to gastrectomy.

## 4 Conclusion

Multifocality in EGC can be a problem for minimally invasive treatment such as endoscopic excision. An adequate preoperative investigation by endoscopy, chromoendoscopy and multidisciplinary approach are very important to plan the best therapeutic strategy in order to reduce the under-treatment risk. Moreover, genetic and molecular features analysis could play a key role. Consistently with the high variability of OS between different endoscopic and surgical approaches in literature, further studies will be needed to evaluate the safety and feasibility of subtotal vs total gastrectomy in this kind of disease, considering that the use of laparoscopic total gastrectomy for gastric cancer remains controversial.

## Declaration of Competing Interest

The authors declare that they have no conflict of interest.

## Sources of funding

No funding has been received for this study.

## Ethical approval

All procedures performed in studies involving human participants were in accordance with the ethical standards of the institutional and/or national research committee and with the 1964 Helsinki declaration and its later amendments or comparable ethical standards.

## Consent

Informed consent was obtained from the patient prior to surgical procedure, figures used anonymous data.

## Author contribution

Conception and design, acquisition, analysis and interpretation: T. Z., M. A., G. S., F. C.;

Writing and revising it critically: T. Z., M. A., G. S., F. C.; all authors contributed to this paper for the final approval of the final version.

## Registration of research studies

NA.

## Guarantor

Tommaso Zurleni, MD.

## Provenance and peer review

Not commissioned, externally peer-reviewed.
